# The Association between Epidermal Growth Factor Receptor (*EGFR*) Gene Polymorphisms and Lung Cancer Risk

**DOI:** 10.3390/biom8030053

**Published:** 2018-07-13

**Authors:** Nabil A. Bashir, Entesar S. Ragab, Omar F. Khabour, Basheer Y. Khassawneh, Mahmoud A. Alfaqih, Jafar A. Momani

**Affiliations:** 1 Department of Physiology and Biochemistry, Faculty of Medicine, Jordan University of Science and Technology, Irbid 22110, Jordan; nbashir@just.edu.jo (N.A.B.); hedaia2008x@hotmail.com (E.S.R.); maalfaqih@just.edu.jo (M.A.A.); 2 Department of Medical Laboratory Sciences, Faculty of Applied Medical Sciences, Jordan University of Science and Technology, Irbid 22110, Jordan; 3 Department of Internal Medicine, Faculty of Medicine, Jordan University of Science and Technology, Irbid 22110, Jordan; basheerk@just.edu.jo; 4 Respiratory Medicine Division, King Hussein Medical Center, Amman 11733, Jordan; jafarmom@yahoo.com

**Keywords:** *EGFR*, polymorphism, haplotype, lung cancer, Jordan

## Abstract

Lung cancer is the leading cause of cancer death globally. The epidermal growth factor receptor (EGFR) plays an important role in cell proliferation and signaling. In this study, we examined the association between *EGFR* gene polymorphisms and lung cancer risk among the Jordanian population. A total of 129 patients with primary lung cancer and 129 matched healthy controls were recruited into this study. *EGFR* rs712829, rs712830, rs2072454, and rs11543848 single nucleotide polymorphisms (SNPs) were genotyped to test for their association with lung cancer risk. A significant association was observed between the rs712829 SNP and lung cancer risk (*p* < 0.05) where the GG + GT genotypes were higher in lung cancer patients when compared to controls. In addition, no association was detected between rs712830, rs2072454, and rs11543848 SNPs and lung cancer risk. When patients were stratified according to the lung cancer type, a significant association was detected between both rs712829 and rs2072454 and adenocarcinoma lung cancer (*p* < 0.05). Haplotype analysis of all four SNPs showed a significant association between the TCCG haplotype and both lung cancer and the adenocarcinoma subtype (*p* < 0.001). In conclusion, *EGFR* rs712829, rs2072454 SNPs, and TCCG haplotypes are associated with a risk of lung cancer among Jordanians. Since genetic associations are affected by the genetic background of populations, more studies in other Arab populations are required to confirm the present findings.

## 1. Introduction

With over 1.3 million deaths per year, lung cancer is by far the leading cause of death among men and women worldwide [1]. It usually starts in the lung. This is known as primary lung cancers and then the cancer spreads into neighboring organs [2]. Among the many risk factors known to be associated with increased lung cancer risk, tobacco smoking appears to have the strongest association [3,4]. Nonetheless, recent studies showed that about 10% to 15% of lung cancer cases occur in people who have never smoked, which indicates that factors such as genetic polymorphisms may play a role in determining disease risk [5]. Most current statistics estimated that about 8% of lung cancer cases are solely due to inherited factors [6]. The contribution of hereditary factors in determining disease risk is further supported by the fact that the total risk of lung cancer is increased 2.4 fold in people who are direct relatives of lung cancer patients [7].

The discovery of genetic markers associated with increased susceptibility to lung cancer is an active area of research [8]. The epidermal growth factor receptor (EGFR) is a tyrosine kinase receptor encoded by a gene located on the short arm of chromosome 7 [9]. It belongs to the ErbB family and plays a significant role in regulating many different signaling pathways that include cell proliferation [9]. EGFR is frequently overexpressed in many cancers including non-small cell lung cancer (NSCLC) [10]. Furthermore, EGFR overexpression is a marker showing a poor prognosis in lung cancer and other cancer types [11]. The role of EGFR in mediating lung cancer pathogenesis is further reinforced by the fact that several histological types of lung cancer respond to therapeutics that inhibit EGFR and/or its downstream effectors [11]. These observations suggest that the *EGFR* gene may harbor mutations and/or polymorphisms that increase the susceptibility to lung cancer [12]. Most of these mutations/polymorphisms are in the catalytic kinase domain, which increases EGFR phosphorylation activity. However, any mutation or polymorphism that affects EGFR expression or activity may theoretically modify lung cancer risk [13,14]. The relationships between *EGFR* gene polymorphisms and the risk of lung cancer from previous studies are still controversial [6,12,15,16,17,18,19]. In this study, we examined the association between a number of SNPs located in different areas of the *EGFR* gene known as rs712829, rs712830, rs2072454, and rs11543848. The risk of lung cancer in the Jordanian population is an example of the Arab populations.

The rs712829 and rs712830 SNPs are located in the promoter region and could affect the expression of the *EGFR* gene and, therefore, the activity of the receptor [18]. The rs2072454 SNP is expressed in the ligand binding site of the EGFR protein while the rs11543848 SNP is located in the extracellular domain, which controls the binding arm [20,21]. The results will enhance our knowledge with respect to the contribution of *EGFR* SNPs to the development of lung cancer and may serve as potential genetic markers for predicting lung cancer risk.

## 2. Materials and Methods

### 2.1. Subjects

This was a case-control study designed to assess the association between four SNPs and lung cancer risk in the Jordanian population. A total of 130 patients with primary lung cancer and 130 matched healthy controls were recruited for the study from the King Abdullah University Hospital and the Jordanian Royal Medical Services from March 2016 until April 2017. Clinical data were collected from the patients’ medical records while demographics were collected through a questionnaire. The study was approved by the Institutional Review Boards of Jordan University of Science and Technology and the Jordanian Royal Medical Services. Informed consents were obtained from all participants after a full explanation of the study objectives and procedures. Primary lung cancer was diagnosed by a respiratory consultant and was confirmed by a histopathology examination. All patients were invited to participate regardless of the type of lung cancer or the stage.

### 2.2. Blood Sampling

Blood samples (3 mL each) were collected in EDTA tubes and stored at −20 °C for DNA extraction and genotyping.

### 2.3. Genomic DNA Extraction

DNA was extracted from whole blood using a Puregene^®^ Blood Core Kit B (Lot No. 8510944, Germantown, MD, USA). The extraction process was performed according to the manufacturer’s instructions. Assessment of the DNA yield was completed by NanoDrop (Thermo Scientific, ND-2000 UV-Vis Spectrophotometer, Waltham, MA, USA). The extracted DNA was stored −20 °C until further use in genotyping of the different SNPs [22].

### 2.4. Genotyping Analysis

Information about *EGFR* gene polymorphisms were obtained from the public SNP database (National Institute of Health, Bethesda, MD, USA; http://www.ncbi.nlm.nih.gov/SNP). EGFR SNPs called rs2072454, rs11543848, rs712829, and rs712830 were analyzed. The sequences of the primers used for PCR amplification are shown in Table 1. In order to genotype rs2072454 by restricting fragment length polymorphism (RFLP), a recognition site for *Bstu1* was introduced into the PCR product. This was achieved by designing a reverse primer with a C at the 3′ end of the primer instead of a T [15]. The rs11543848 SNP was also analyzed by PCR, which was followed by RFLP using the *BstNI* enzyme (Biolabs, Ipswich, MA, USA) previously described [20]. Amplification of target sequences was performed using a ready to use master mix (Promega, Madison, WI, USA). PCR conditions are shown in Table 1. Verification of the size of the PCR product was performed by electrophoresis using a 2.5% agarose gel stained with ethidium bromide. The PCR bands were visualized under UV light using a gel documentation system (Gel Doc 2000, Bio-Rad, Hercules, CA, USA). Digestion reaction conditions were performed per manufacturer recommendations.

With respect to rs712829 and rs712830, they were genotyped by direct DNA sequencing of an amplified PCR product that contains the SNP. The genotyping was completed following PCR amplification and confirming the size of the product. The resulting amplicon was purified using an EZ-10 Spin Column PCR Product Purification Kit (Bio Basic INK, Toronto, ON, Canada). Sequencing was performed using BigDye technology Terminator kits on an Applied Biosystems 3130/3130xl analyzer. ChromasPro software (South Brisbane, Australia) version 1.7.4 was used to analyze the electropherogram files.

### 2.5. Statistical Analysis

Data analysis was performed using the statistical package for social studies (SPSS) version 21 (Armonk, NY, USA). Frequencies were compared between lung cancer patients and controls using the Pearson chi-square test. The SNPStats software tool was used to conduct the haplotype analysis (http://bioinfo.iconcologia.net/SNPstats). The null hypothesis was rejected if the *p*-value < 0.05.

## 3. Results

Table 2 shows the demographics of the study participants. Lung cancer patients were matched with lung cancer free controls in age, sex, and smoking status (*p* > 0.05). About 80% of the patients were older than 50 years with a mean age of 61.8 ± 0.9 years. The majority (>80%) were males and smokers/ex-smokers. Histopathological examination showed that 90% of the patients had non-small cell lung cancer (NSCLC) and 10% had small cell lung cancer (SCLC). About one third (33%) of NSCLC were squamous carcinoma, 30% were with a non-specific definition, 26% were adenocarcinoma, and 1% were large cell lung carcinoma.

Table 3 shows the distribution of the different genotype categories of all examined *EGFR* SNPs. All SNPs were in the Hardy-Weinberg equilibrium (*p* > 0.05). For rs712829, there was a significant decrease in TT frequency among lung cancer patients (17%) when compared to controls (27%, *p* < 0.05). On the other hand, the GG + GT genotype frequencies were significantly higher (*p* < 0.05) in lung cancer patients (83%) when compared to controls (73%). With respect to rs712830, rs2072454, and rs11543848, there were no significant differences in the genotype or in the allele frequencies between patients and controls (*p* > 0.05, Table 3).

Table 4 shows the haplotype analysis for rs712829, rs712830, rs2072454, and rs11543848 SNPs. The TCTA haplotype pattern (0.2547) was the most frequent and was followed by the GCTG pattern (0.1941). The result showed no significant association between haplotypes and lung cancer risk except for the TCCG haplotype with a frequency of 0.0408 among the examined population (*p* < 0.001).

The contribution of hereditary factors to lung cancer risk appears to be different among the different histological types with the risk of having adenocarcinoma being predominantly determined by genetic rather than environmental factors [23,24]. Therefore, the frequencies of all examined SNPs were compared between adenocarcinoma lung cancer patients (*n* = 43) and the lung cancer-free control group (Table 5). Results showed significant association between adenocarcinoma with two of the examined SNPs known as rs712829 or rs2072454 (*p* < 0.05). For rs712829, the frequency of the TT genotype was lower in the lung cancer-free controls compared to lung cancer of the adenocarcinoma type while the frequencies of the GG + GT genotypes were higher in the adenocarcinoma type (*p* < 0.05). With respect to rs2072454, the frequency of the TT genotype was higher in adenocarcinoma compared to lung cancer-free controls. However, the frequency of the CT genotype was lower in lung cancer patients with the adenocarcinoma group compared to the lung cancer-free controls (*p* < 0.05). Haplotype analysis (Table 6) showed significant association between the TCCG haplotype of rs712829, rs712830, rs2072454, and rs11543848 SNPs and adenocarcinoma with a frequency of 0.0629 in the population (*p* < 0.001).

## 4. Discussion

In the current investigation, we examined the association between four SNPs in the *EGFR* gene (rs712829, rs712830, rs2072454, and rs11543848) and lung cancer risk. Results showed significant association between rs712829 and primary lung cancer risk when lung cancers of all histological types were included in the analysis. When testing the association of all four SNPs with lung cancer of the adenocarcinoma type only, significant associations were observed between the risk of adenocarcinoma lung cancer and either rs712829 or rs2072454. Lastly, haplotype analysis of all four SNPs with lung cancer risk showed a strong association between the TCCG haplotype and lung cancer. A significant association of the same haplotype with lung cancer was also observed when the analysis was performed on lung cancer patients of the adenocarcinoma type only.

The results showed a significant association between rs712829 SNP and lung cancer risk. This SNP is located in the promoter region of the *EGFR* gene and was shown in previous studies to modify the expression of the *EGFR* gene [25]. According to the results of our analysis, the frequency of the TT genotype was higher in the lung cancer free group compared to patients diagnosed with lung cancer. Accordingly, the presence of the T allele may be protective against lung cancer. Several models were tested to identify the genetic model of inheritance that best explains the protective effect of the T allele in the examined population. The analysis showed that the best genetic model for explaining the variation in the genotype frequencies of rs712829 between the cases and control groups is a recessive model of inheritance. In this model, the presence of two copies of the T allele are needed to reduce the risk of lung cancer. The above model also implies that the GG genotype of rs712829 significantly increases the risk of lung cancer in the Jordanian population. Moreover, a significant association was also found between rs712829 and the risk of lung cancer of the adenocarcinoma subtype. A previous study reported an association between the rs712829 TT genotype and the pleural metastasis of lung adenocarcinoma in the Chinese population [26]. In addition, a lack of association between the rs712829 SNP and lung cancer was reported in a United States population [18] and a Japanese population [19]. Inconsistency was reported between the rs712829 SNP and clinical outcomes of lung cancer. For example, rs712829 polymorphism has been shown to influence the response to EGFR-TKIs therapy in Chinese and Korean lung cancer patients [27,28] while a lack of association was reported in the Japanese population [19]. The inconsistent finding related to the association between rs712829 and lung cancer can be explained by the differences in the genetic background of the studied populations. For example, it is possible that other polymorphisms are present in the region of the *EFGR* gene in the studied populations that modulate the effect of the rs712829 SNP.

The results showed an association between *EGFR* rs2072454 SNP and adenocarcinoma lung cancer with the TT genotype, which exhibited a link to the disease. Consistent with this finding, rs2072454 has been shown to be associated with risk for head and neck squamous cell carcinoma [29] and gastric cancer [28]. However, a lack of association between the rs2072454 SNP and lung cancer was found in the Korean population [15].

Based on ethnicity, the differences in associations between genetic variations and diseases are very common. This includes the role of *LOXL* rs1048661 SNP in the development of exfoliation glaucoma (XFG). The G allele of the rs1048661 SNP has been shown to be associated with the risk of XFG in European populations and it has been shown to protect against the disease in Asian populations [30]. Therefore, the observed opposite effect of EFGR rs712829 SNP in the Jordanian population compared to the Chinese is not surprising and the literature is rich in such examples [31,32]. However, finding the mechanisms behind such opposite effects, based on ethnicity, requires more investigations. In addition, further studies that include direct measurement of EFGR protein levels according to different genotypes are required to understand the discrepancies observed in the different studies.

The results showed a lack of association between rs712830 and rs11543848 of the *EGFR* gene and lung cancer. The rs712830 (-191C/A) is located in the promoter region of the *EGFR* gene while rs11543848 SNPs are located in the extracellular domain. However, these SNPs do not cause critical changes on the protein level of *EGFR*. A recent meta-analysis study concluded that rs11543848 is not associated with cancer risk [33]. The lack of association between rs712830 SNP and lung cancer was also observed in the American population [18].

In the current study, haplotype analysis was also performed to examine any possible role of the examined SNPs with adenocarcinoma lung cancer. The result showed a strong association between the TCCG haplotype of rs712829, rs712830, rs2072454, and rs11543848 and lung cancer/adenocarcinoma. Previous studies have shown that haplotypes of *EGFR* SNPs are associated with non-small cell lung cancer [34], lung adenocarcinoma [17], glioma [35], and glioblastoma [36]. This result sheds light on the importance of studying the association of haplotypes of more than one SNP and disease risk. It is also important to consider haplotypes as important biomarkers for lung cancer prediction in the Jordanian population. More investigations are needed to examine the effect of these haplotypes on EGFR protein levels to enhance our knowledge about their impacts on EGFR signaling pathways.

Lastly, the results showed that about 82% of lung cancer patients included in the study were smokers or ex-smokers. This result is consistent with the fact that smoking plays an important role in the etiology of lung cancer [5]. The increasing risk of lung cancer in non-smokers could be due to multiple possible causes such passive smoking, exposure to air pollution, and genetic predisposition. These possibilities were not explored in the current study and are worth examining in future investigations.

One of the limitations of the current study is the relatively small sample size. In addition, *EGFR* contains other SNPs that were not investigated in the current study and might be associated with lung cancer among Jordanians. In addition, the relationships between *EGFR* SNPs and clinical factors of lung cancer such as clinical prognosis, stage, and metastatic status were not examined. Future studies that include more patients, other SNPs in *EGFR*, and clinical factors of lung cancer are strongly recommended. Lastly, future studies that link *EGFR* SNPs to EGFR protein expression in lung tissues are essential for understanding the mechanisms by which *EGFR* SNPs contribute to lung cancer [37].

## 5. Conclusions

In conclusion, EGFR rs712829 and rs2072454 SNPs may be associated with lung cancer among Jordanians. In addition, TCCG haplotype of rs712829, rs712830, rs2072454, and rs11543848 was strongly associated with lung cancer/adenocarcinoma. More studies are required to confirm the present findings.

## Figures and Tables

**Table 1:** The primer pairs for each SNP and their characteristics.

SNP	Primer Pairs	PCR Conditions *	Product Size
**rs2072454**	5′CATAAATGCGAAGAGCACATGCATCCTTC′3 5′CTGACTATGTCCCGCCACTGGATGCTCTCCG′3	35 cycles: 94 °C for 1 min, 66 °C for 45 s and 72 °C for 45 s.	166
**rs11543848**	5′TCTGCCATGCCTTGTGCTCC′3 5′CCTGGAGCCTTATTTTTGATC′3	35 cycles: 94 °C for 1 min, 56 °C for 45 s and 72 °C for 45 s.	171
**rs712829 & rs712830**	5′GAGCTAGACGTCCGGGCA′3 5′GCTCTCCCGATCAATACTGGA′3	35 cycles: 94 °C for 1 min, 64 °C for 60 s and 72 °C for 1 min.	240

* All reactions were preceded by 10 min of 94 °C and ended by 5 min of 72 °C.

**Table 2:** Characteristics of study participants.

Variable	Lung Cancer Patients (*n* = 129)	Controls (*n* = 129)	*p*-Value
**Age (years)**	61.8 ± 0.9	62.0 ± 0.8	0.94
**Gender**			
Males	105 (81.4%)	105 (81.4%)	1.0
Females	24 (18.6%)	24 (18.6%)	
**Smoking status**			
Smokers	91 (70.5%)	92 (71.3%)	0.92
Ex-smokers	11 (8.5%)	11 (8.5%)	
Non-smokers	22 (17%)	23 (17.8%)	
Unknown	5 (4%)	3 (2.3%)	

**Table 3:** The distribution of genotypes of *EGFR* SNPs in the patient and the control groups.

SNP ID	Genotypic/Allele Pattern	Patients Frequencies	Controls Frequencies	*p*-Value
**rs712829**	GG	36 (29%)	29 (22%)	<0.05
	TT	21 (17%)	35 (27%)	
	GT	67 (54%)	65 (50%)	
**rs712830**	CC	116 (93%)	121 (94%)	0.75
	CA	9 (7%)	8 (6%)	
**rs2072454**	CC	19 (15%)	15 (12%)	0.60
	TT	60 (47%)	57 (44%)	
	CT	50 (39%)	57 (44%)	
**rs11543848**	AA	24 (19%)	26 (20%)	0.90
	GG	16 (13%)	18 (14%)	
	GA	87 (69%)	85 (66%)	

**Table 4:** Association between SNP haplotypes and lung cancer risk.

Haplotype	Rs29	Rs30	Rs54	Rs48	Frequency	Odd Ratio (95% CI)	*p*-Value
1	T	C	T	A	0.237	1.00	---
2	G	C	T	G	0.1926	1.20 (0.31–4.61)	0.79
3	G	C	T	A	0.1534	0.25 (0.03–21.82)	0.17
4	T	C	T	G	0.1374	0.86 (0.13–5.54)	0.88
5	G	C	C	A	0.0838	0.31 (0.03–3.30)	0.33
6	T	C	C	A	0.0776	2.49 (0.24–26.01)	0.45
7	G	C	C	G	0.0528	0.00 (inf-inf)	1
8	T	C	C	G	0.0416	996,206 (996,198–996,214)	<0.001
9	G	A	T	G	0.0233	2.00 (0.11–35.14)	0.64

Note: Rs29: rs712829, Rs30: rs712830, Rs54: rs2072454, and Rs48: rs11543848.

**Table 5:** The genetic pattern frequencies of rs712829, rs712830, rs2072454, and rs11543848 in patients with an adenocarcinoma subtype and controls.

Genotypes/Allele Pattern	Patients	Controls	*p*-Value
**For rs712829**			
GG	12 (28%)	29 (22%)	0.020
TT	4 (9%)	35 (27%)	
GT	27 (63%)	65 (50%)	
**For rs712830**			
CC	41 (95%)	121 (94%)	0.670
CA	2 (5%)	8 (6%)	
**For rs2072454**			
CC	6 (14%)	15 (12%)	0.027
TT	26 (60%)	57 (44%)	
CT	11 (26%)	57 (44%)	
**For rs11543848**			
AA	8 (19%)	26 (20%)	0.073
GG	2 (5%)	18 (14%)	
GA	33 (77%)	85 (66%)	

Note: Rs29: rs712829, Rs30: rs712830, Rs54: rs2072454 and Rs48: rs11543848.

**Table 6:** Association between SNPs haplotypes and adenocarcinoma lung cancer risk.

Haplotype	Rs29	Rs30	Rs54	Rs48	Frequency	Odd Ratio (95% CI)	*p*-Value
1	T	C	T	A	0.2118	1.00	---
2	G	C	T	A	0.1748	1.00 (0.31–3.24)	1
3	G	C	T	G	0.1552	0.93 (0.34–2.55)	0.9
4	T	C	T	G	0.1228	1.38 (0.32–5.96)	0.66
5	G	C	C	G	0.0953	1.08 (0.31–3.67)	0.91
6	T	C	C	A	0.0794	1.46 (0.33–6.43)	0.62
7	G	C	C	A	0.0681	0.69 (0.15–3.15)	0.63
8	T	C	C	G	0.0629	168,507 (168,498–168,517)	<0.001
9	G	A	T	G	0.0156	0.71 (0.08–6.39)	0.76

Note: Rs29: rs712829, Rs30: rs712830, Rs54: rs2072454 and Rs48: rs11543848.
